# Defining Training in Critical Care Cardiology: What Is the “Gold Standard?”∗

**DOI:** 10.1016/j.jacadv.2024.100849

**Published:** 2024-02-07

**Authors:** Brandon M. Wiley, Emily K. Zern

**Affiliations:** Division of Cardiology, Los Angeles General Medical Center, Keck School of Medicine of University of Southern California, Los Angeles, California, USA

**Keywords:** critical care cardiology, cardiac intensive care unit, training

**Figure:**
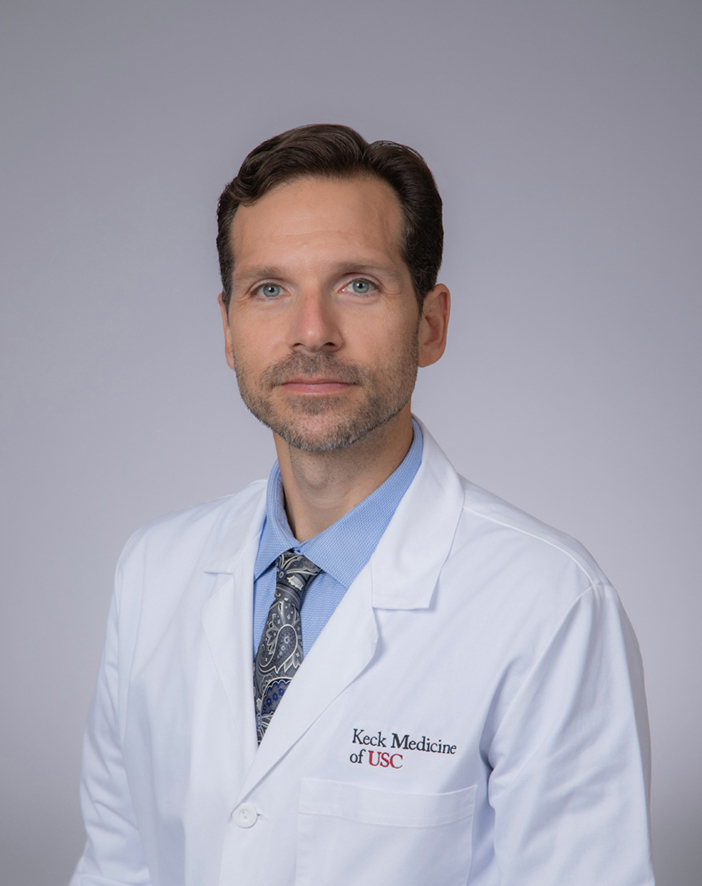

The modern cardiac intensive care unit (CICU) is responsible for array of cardiac patients with concomitant noncardiac multiorgan system dysfunction and complex shock states.^1^ Such a breadth and depth of CICU care mandates a specialized comprehensive staffing model that includes cardiac intensivists, advanced practice practitioners, nurses, pharmacists, respiratory therapists/perfusionists, trainees, and support staff.^2^ However, despite growing data supporting the value of specialized cardiac intensivists throughout the spectrum of cardiac and cardiothoracic surgical care in CICUs,^3^^,^^4^ there is an insufficient supply of cardiologists with the requisite training in cardiac critical care (CCC) to meet the demands of current clinical practice.

In this issue of *JACC: Advances*, Vallabhajosyula et al.^5^ present a scoping review of the published training pathways in CCC. The authors’ found a heterogeneous mix of training options that they condensed into: 1) the traditional pathway of separate and sequential fellowship training programs in cardiovascular medicine (CV) and critical care medicine (CCM) to achieve American Board of Internal Medicine (ABIM) board eligibility in both specialties; or 2) elective rotations in CCM during specialty or subspecialty training in CV, interventional cardiology (IC), or advanced heart failure (HF) leading to a non-ABIM certificate in CCC.^5^ As the ABIM requirements for CV and CCM require at least 4 years of total fellowship training for board eligibility in both, there is interest in creating an holistic integrated curriculum in CCC spanning the length of CV fellowship training, though a blueprint for achieving such a program that will meet both American College of Graduate Medical Education and ABIM guidelines has not been established.

After synthesizing available literature, the authors suggest that the integration of focused critical care training into the existing framework of cardiology fellowship programs (CV, IC, or HF) would be a suitable solution to help mitigate the current shortage of CCC physicians and to reduce the total training time when compared to traditional pathways that combine standalone fellowships in cardiology and CCM. However, the integration of focused CCC training into existing cardiology fellowships would not yield eligibility for ABIM CCM certification. Instead, fellows that chose to enrich their cardiology training with CCC experience would achieve non-ABIM certifications in CCC, stratified to reflect the amount of additional CCC training that was completed. Ultimately, the non-ABIM CCC certifications achieved by each trainee could be used to match their desired acuity level of CICU practice (Level 1, Level 2, and Level 3 centers).

We read the authors’ scoping review with great interest, as addressing the shortage of trained cardiac intensivists is a principal challenge that threatens the continued growth of CCC within the current cardiology and critical care clinical landscape. The authors’ findings speak to the need for a consensus gold-standard pathway for CCC training that can provide a consistent pipeline of cardiologists entering clinical practice with the defined critical care expertise to staff contemporary CICUs. When considering the scope of the field of CCC and the career pathway of trainees, it is important to note that the practice of CCC is evolving. For example, the traditional dividing lines between the CICU and the cardiothoracic surgical intensive care unit are becoming less defined, particularly with the growing utilization of mechanical circulatory support (MCS) therapies including extracorporeal membrane oxygenation (ECMO).^6^^,^^7^ The development of large comprehensive “heart centers” or integrated cardiac units are redefining “cardiac critical care.” The training pathways for CCC should therefore adequately equip those pursuing the field to care for patients across the spectrum of cardiovascular disease, including advanced HF, MCS/ECMO, and postcardiotomy patients, of which CCC physicians may not be primarily attending but must collaborate with colleagues from other specialties. Partial CCC training (analogous to Cardiology Core Cardiovascular Training Statement 1 or 2) or hybrid models within HF or IC that do not achieve ABIM CCM board certification may successfully prepare trainees to fit within their specific institution’s needs (or those of similar institutions). However, this strategy may ultimately limit the trainees’ marketability and preparedness elsewhere, particularly in the competitive landscape of comprehensive cardiac centers in which the CICU may be under the direction of a department or division of critical care. Cardiologists with partial CCC training may find themselves competing for positions in the CICU with colleagues who have more in-depth critical care experience such as pulmonary, surgical, or anesthesia critical care.

Defining the standard for CCC training is crucial for the future of CCC practice within the landscape of critical care. CCC trainees not only need to develop the clinical expertise to manage critically ill cardiac patients, but they also need to be recognized by the critical care community as critical care physicians. CV fellows need a clear definition of the CCC training process and reassurance that their efforts will yield marketable skills and a clinical position post-fellowship. Therefore, gold-standard CCC training should: 1) be sufficient to achieve ABIM CCM certification to provide validity to the training; and 2) specify procedural and clinical competencies relevant to the modern field of CCC to confirm that a board certification does not just represent perceived competence in the field. At this time, there is no guidance within CCM on the minimum number of procedures performed analogous to the Cardiology Core Cardiovascular Training Statement level I-III procedural requirements. Though generalized critical care competencies are defined by the ABIM (ie, competence in renal replacement therapies and mechanical ventilation), more specified procedural and clinical requirements may be essential for the field of CCC. Defining the benchmarks for cases of MCS/ECMO management, critical care ultrasound, time in the operating room with cardiac anesthesia, and time spent caring for postcardiothoracic surgical patients, in addition to required numbers of critical care procedures (bronchoscopy, airway management, chest thoracostomy) would provide CCC trainees with transparent competencies when applying for faculty positions. Broad training across the spectrum of cardiovascular disease and critical care may help facilitate acceptance of CCC not only as part of, but as leaders of, the critical care community. As comprehensive cardiac centers develop all-encompassing CICUs, it is imperative that cardiology-trained cardiac intensivists remain at the forefront of this competitive landscape, with the clinical competence to work alongside physicians from other critical care backgrounds.

Though an additional year of training in CCM may be unattractive for those also pursuing HF or IC, it is possible that specific institutions may create integrated critical care programs over a total of 4 or 5 years of fellowship training through collaboration with existing pulmonary or surgical critical care fellowships. These collaborative approaches are attractive to leverage the existing training framework and provide multidisciplinary critical care education, though they should not shortcut the requirements of a standardized CCC program or compromise ABIM board certification.

The authors should be commended for performing a thoughtful scoping review that serves to: 1) define the heterogeneity in current training pathways in CCC; and 2) highlight the need to find balance between optimal training for CCC physicians and the need for flexibility to address the acute and persistent need for physician staffing within existing CICUs. Of the studies included in this review, the vast majority (80%, 16/20) were opinion pieces, and only 10% (2/20) were original research pieces utilizing survey methodology. This speaks to the paucity of data regarding existing pathways within CCC, despite the ever-growing demand to define the field.

Future studies may prospectively collect data regarding the clinical experiences and procedural numbers of graduating CCC fellows from existing programs and evaluate this against the clinical scope of subsequent faculty positions for these graduates. Such data may better inform and refine the development of a standardized CCC training pathway, with the collective goal of generating a sufficient, broadly trained cardiac intensivist workforce for the modern CICU.

## Funding support and author disclosures

The authors have reported that they have no relationships relevant to the contents of this paper to disclose.
